# Progesterone - new therapy in mild carpal tunnel syndrome? Study design of a randomized clinical trial for local therapy

**DOI:** 10.1186/1749-7221-5-11

**Published:** 2010-04-26

**Authors:** Paolo Milani, Mauro Mondelli, Federica Ginanneschi, Riccardo Mazzocchio, Alessandro Rossi

**Affiliations:** 1 Dept. Neurological, Neurosurgical and Behavioural Sciences, Neurophysiology Clinic Section, University of Siena, Siena, Italy; 2 Service de Physiologie Explorations Fonctionnelles, Hôpital Lariboisière, AP-HP, 2 rue Ambroise-Paré, 75010 Paris, France; 3 Université Paris 7 Denis-Diderot, 2 rue Ambroise-Paré, 75010 Paris, France; 4 EMG Service, Local Health Unit 7, Siena, Italy

## Abstract

**Background:**

Local corticosteroid injection for carpal tunnel syndrome (CTS) provides greater clinical improvement in symptoms one month after injection compared to placebo but significant symptom relief beyond one month has not been demonstrated and the relapse of symptoms is possible.

Neuroprotection and myelin repair actions of the progesterone was demonstrated in vivo and in vitro study.

We report the design of a randomized controlled trial for the local injection of cortisone versus progesterone in "mild" idiopathic CTS.

**Methods:**

Sixty women with age between 18 and 60 years affected by "mild" idiopathic CTS, diagnosed on the basis of clinical and electrodiagnostic tests, will be enrolled in one centre. The clinical, electrophysiological and ultasonographic findings of the patients will be evaluate at baseline, 1, 6 and 12 months after injection.

The major outcome of this study is to determine whether locally-injected progesterone may be more beneficial than cortisone in CTS at clinical levels, tested with symptoms severity self-administered Boston Questionnaire and with visual analogue pain scale.

Secondary outcome measures are: duration of experimental therapy; improvement of electrodiagnostic and ultrasonographic anomalies at various follow-up; comparison of the beneficial and harmful effects of the cortisone versus progesterone.

**Conclusion:**

We have designed a randomized controlled study to show the clinical effectiveness of local progesterone in the most frequent human focal peripheral mononeuropathy and to demonstrate the neuroprotective effects of the progesterone at the level of the peripheral nervous system in humans.

## Introduction

Fifty years after its widespread recognition, a significant minority of patients with carpal tunnel syndrome continue to experience poor outcomes from treatment. Much of the current treatment is supported by inadequate or nonexistent evidence. Surgical decompression, often considered the definitive solution, leads to positive results in 75% of the cases, but leaves 8% of patients worse than before [1].

Open release is the preferred surgical procedure. Some patients referred failure to relieve symptoms after decompression surgery, and reoperation is sometimes necessary. This is consequence of incomplete release of the flexor retinaculum, scarring around the median nerve, or incorrect diagnosis [2-4]. Open release is not without complications, these produce symptoms different from those present before surgery and can be very disabling and difficult to treat. The "major" complications are rare and consists in lesion of the recurrent motor branch, severance of the palmar cutaneous branch of the median nerve or of palmar terminal branches of the median or ulnar nerves with or without neuroma, bowstringing of the flexor retinaculum, tendon or artery injuries, reflex sympathetic dystrophy. The "minor" complication are more frequent (pillar pain, loss of grip, scar tenderness or hypertrophy, wound infection, trigger finger) [5-7].

Pillar pain and loss of grip are temporary and spontaneously disappear within about 3 months, even if some authors reported the persistence of pillar pain and scar tenderness even after 2 years of follow-up [8]. Endoscopic and limited-incision techniques seem to have fewer complications than classical open surgery, but meta-analysis study was inconclusive on which is the best surgical approach [9]. However endoscopic technique provides faster relief of pain, more rapid improvement in functional abilities and earlier return to work [10].

The nonsurgical interventions with clear benefit are neutral-angle wrist splinting (with a success rate of 37%), and steroids, which show better effects when administered by local injection than orally. The initial positive response rate to injection is 70%, but there are frequent relapses as demonstrated by the 12 studies included in the last Cochrane Review [11].

We have designed a randomised controlled study, realized according to apt criteria for the appraisal of the effectiveness of every new therapeutic strategy, in order to demonstrate the clinical effectiveness of local progesterone in the most frequent human focal peripheral mononeuropathy and the neuroprotective effects at the level of the peripheral nervous system in humans.

## Background

Carpal tunnel syndrome (CTS) results from the compression of the median nerve at the wrist. The typical symptoms are paraesthesiae (numbness, tingling) and pain in the hand distribution of the median nerve, more often occurring during night or in early morning waking up the patients. There may be also sensory loss and hand weakness and clumsiness causing difficulties in daily activities. The prevalence of median nerve symptoms and electrophysiological median neuropathy in the general population of Maastricht was 3.4% for women and 0.6% for men, but another 5.8% of all adult women has undetected CTS [12]. The annual average crude incidence in the Siena area is 329.4/100000 person-years with a highest incidence in range age 50 to 59 years [13].

The severity of CTS ranges from mild to severe. In mild CTS, focal disturbance to myelin is the dominant factor and indeed paranodal demyelination has been documented [14,15], whereas only with more severe nerve compression do demyelination and Wallerian degeneration occur [16,17]. Consequently, patients with mild CTS generally report intermittent symptoms can cause permanent loss of sensation and partial paralysis in abduction and opposition of the thumb, whereas severe CTS can cause permanent loss of sensation and paralysis in abduction and opposition of the thumb.

## The Problem

CTS can be treated with surgery or conservative options. There are many conservative treatments commonly used in mild and moderate CTS: oral and local steroids, non steroidal anti-inflammatory drugs (NSAIDs), diuretics, pyridoxine, wrist splints, physical therapy, therapeutic exercises and manipulations [18,19].

From the reported data it can conclude the following: (1) locally injected steroids produce significant improvement [20], even if this is temporary (strong evidence, level 1) at both low and high doses, though they may give side-effects (strong evidence, level 1); (2) vitamin B6 is ineffective (moderate evidence, level 2) whereas NSAIDs and diuretics are effective (limited evidence, level 3). Among physical treatments there are conflicting evidences. Only splints are effective, especially if used full-time (moderate evidence, level 2).

The local corticosteroid injection is the principal alternative to surgery. In one randomized comparison of management by injection or surgery, equivalent success rates were found at 1-year follow-up [21] but an open follow-up study of this cohort of injected patients showed that injected patients continue to experience relapse of symptoms up to at least 7 years after injection, whereas, in surgically treated patients, relapse after 1 year is very rare. Although popular in rheumatological practice, this intervention has been scorned by most surgeons. Known risks, such as cutaneous atrophy and depigmentation, tendon rupture, and median nerve injury from inadvertent intraneural injection, have been given great prominence, and the therapeutic effect has generally been considered to be of lower quality than that provided by surgery and temporary in nature. Same surgeons have argued that steroid injection merely masks the symptoms, whereas median nerve degeneration continues to progress to a point where subsequent surgical outcome is prejudiced [1]. In Cochrane study local corticosteroid injection for CTS provides greater clinical improvement in symptoms one month after injection compared to placebo. Significant symptom relief beyond one month has not been demonstrated [11]. Interestingly the improvement of nerve conduction studies found already 1 month after treatment, remaining so until at 6 months [22] but spontaneous improvement of neurophysiologic measurements in CTS has been demonstrated, but only at 10 and 15 months follow-up [23].

In conclusion the anti-inflammatory and anti-edema effects of the corticosteroid are limited at 1 month and in CTS present only a symptomatic effectiveness.

## The Solution

Schwann cells, the myelinating glial cells in the peripheral nervous system, synthesize progesterone in response to a diffusible neuronal signal [24]. In peripheral nerves, the local synthesis of progesterone plays an important role in the formation of myelin sheaths [25,26]. This has been shown in vivo, after cryolesion of the mouse sciatic nerve, and in vitro, in co-cultures of Schwann cells and sensory neurons. After cryolesion axons and their accompanying myelin sheaths degenerate quickly in the frozen zone and the distal segments (Wallerian degeneration). However, the intact basal lamina tubes provide an appropriate environment for regeneration. Schwann cells start to proliferate and myelinate the regenerating fibers after 1 week, and 2 weeks after surgery, myelin sheaths have reached about one third of their final width. In the damaged portion of the nerve, progesterone and pregnenolone (precursor) levels remain high, and even increase 15 days after lesion. The role of progesterone in myelin repair, assessed after 2 weeks, was indicated by the decrease of thickness (number of lamellae) of myelin sheaths when trilostane, an inhibitor of enzyme involved in the pregnenolone to progesterone transformation, was applied to the lesioned nerve. Such an effect was observed in cultures of rat dorsal root ganglia. After 4 weeks in culture, in presence of a physiological concentration of progesterone, the number of myelin segments and total length of myelinated axons are increased enormously.

In addition Schwann cells also express an intracellular receptor for progesterone, which thus functions as an autocrine signaling molecule [27].

Progesterone and its metabolites promote the viability of neurons in the brain, spinal cord and peripheral nervous system. Their neuroprotective effects have been documented in different lesion models, including traumatic brain injury [28], experimentally induced ischemia [29], spinal cord lesions [30,31], and genetic model of motoneuron disease. In experimental diabetic neuropathy [32,33] chronic treatment with progesterone had neuroprotective effects at the neurophysiological, functional, biochemical and neuropathological levels. In this experimental diabetic rats chronic treatment for 1 month with progesterone counteracted the impairment of nerve conduction velocity and thermal threshold, restored skin innervation density, improved Na^+^/K^+ ^ATPase activity and mRNA levels of myelin proteins, such as glycoprotein, peripheral myelin protein 22 (PMP22) and protein zero (P0).

Indeed aging nervous system, that is associated with a reduction in the synthesis of P0 and PMP22, appears to remain sensitive to some of progesterone's beneficial effects [34,35].

Progesterone may promote myelination by activating the expression of genes coding for transcription factors and/or for myelin proteins [36,37] and/or indirectly to regulate myelin formation by influencing gene expression in neurons and may promote neuroregeneration by several different actions by reducing inflammation, swelling and apoptosis, thereby increasing the survival of neurons, and by promoting the formation of new myelin sheaths [27]. Progesterone and its derivates, dihydroprogesterone (5α-DH PROG) and tetrahydroprogesterone (5α-TH PROG), are able to influence the synthesis of myelin proteins under the control of classical receptors (PR, progesterone receptor) and non classical receptors (GABA-A).

PR involvement in the expression of P0 is confirmed by the finding that in cultured rat Schwann cells an antagonist such as mifestone is able to block the stimulatory effect exerted by progesterone and 5α-DH PROG (i.e. classical ligands of PR). This antagonist is also effective in blocking the effect of 5α-TH PROG (i.e. a neuroactive steroid which is able to interact with GABA-A receptor) on P0. Indeed, the activity of the 3α hydroxysteroid dehydrogenase is bi-directional and consequently 5α-TH PROG might be retroconverted into 5α-DH PROG, exerting its effect on P0 via activation of PR [25,38,39].

GABA-A involvement in the expression of PMP22 is confirmed by the finding that in cultured rat Schwann cells a specific GABA antagonist such as bicuculline completely abolishes the stimulatory effect exerted by 5α-TH PROG, while an agonist such as muscimol increase this effect [40].

Finally, progesterone is also well known to have anti-inflammatory property, in view of his effects on pro-inflammatory cytokines [41] and prostaglandins [42]; in addition this hormone has demonstrate capability to decrease edema formation after brain injury [43]. By means of these two properties, progesterone could reduce pain in CTS patients, like the cortisone do [44].

The safety of the progesterone in humans has been demonstrated [28,45].

In summary progesterone can to be a therapeutic opportunity in the myelin neuropathy [46] and the mild CTS is a perfect model of the localized myelin damage.

This is a first randomized clinical trial protocol study in humans for the local therapy in CTS: cortisone versus progesterone.

## Recommendations And Methods

The study is designed as a monocentric randomized clinical trial. The Medical Ethics Committees of the University of Siena approved the study protocol.

### Study population

Patients were enrolled in the study if the symptoms compatible with clinical diagnosis of CTS were confirmed by electrodiagnostic evidence of delay of the distal conduction velocity of the median nerve (for details see respective paragraphs).

Patients who are eligible for participation will be informed about the trial by the neurologist. If they show interest, they will receive written information about the trial with a detailed description of the aim of the study and of the implications of participation. Only subjects able to read, understand and sign the informed consent are included in the study. The information about the trial is also missed to the general practitioner of the patient.

### Clinical criteria

Patients with suspected CTS referred to our electrodiagnostic laboratory to confirm clinical suspicion of CTS will be eligible for including in the trial from 01.06.2008. Patients will be recruited if they will complain at least three months of the following symptoms: nocturnal awaking or activity-related numbness, tingling, burning, pain in the hand distribution of the median nerve according to hand diagram by Katz et al. modified by consensus criteria of classification of the CTS. Only "classic/probable" and "possible" cases will be enrolled [47,48]. Then only patients with "mild" CTS will be successively included in the trial. Mild cases are defined as those patients who complain only symptoms without objective sensory loss, weakness of abduction/opposition of the thumb and hypotrophy/atrophy of thenar eminence, i.e. these patients belonging to stage 1 and 2 of a validated clinical CTS severity scale [49]. Other mandatory inclusion criteria are female gender (because the progesterone is a natural female hormone) and age between 18 and 60 years.

Exclusion criteria are: previous conservative or surgical treatments for CTS, diabetes, connective tissue and thyroid diseases, renal failure, gout, pregnancy, lactation, estrogen drugs, arthritis and deforming arthrosis, onset of CTS symptoms after hand trauma, polyneuropathy, brachial plexopathy, neuropathy of the ulnar nerve at elbow, thoracic outlet syndrome and history of cervical radiculopathy.

Physical examination consisted of evaluating of muscular strength and trophism, sensory function, evocation of deep reflexes and provocative clinical test (Phalen and Tinel signs) will be performed by neurophysiologist before the electrodiagnostic tests.

For subjective evaluation of severity of symptoms, the Boston Questionnaire will be completed by patients just before the injection [50,51]. The questionnaire is divided into two parts. The first part (11 items) evaluates symptoms, and the second part (8 items) evaluates the functional status of the hand. Five answers are possible to each question; they are scored 1 to 5 according to the severity of symptoms or the difficulty of performing a certain activity. Each score is calculated as the mean of the score of individual item. Severe impairment is indicated by a high score.

In addiction visual analogue pain scale (VAS) will be administrated. The patient will be instructed to point to the position on the line between the faces to indicate how much hand pain they are currently feeling. The far left end indicates 'No pain' and the far right end indicates 'Worst pain ever'.

### Electrophysiological criteria

The median and ulnar nerve conduction velocity (NCV) study is performed according to the guidelines of the American Association of Neuromuscular & Electrodiagnostic Medicine for CTS [52]. Surface recording electrodes are placed over the motor point of the abductor pollicis brevis muscle for the median nerve and over that of the abductor digiti minimi for the ulnar nerve. The reference electrode is placed over the tendon. Maximum motor conduction velocity was calculated from elbow to wrist for the median nerve and below-elbow to wrist for the ulnar nerve. Distal motor latency (DML) is measured with a distance of 7 cm between the stimulation point of the nerves at the wrist and the above mentioned muscles. DMLs are measured from the stimulus onset to the initial negative response, and amplitudes are measured from baseline to negative peak. Electrical stimuli are delivered by a constant current stimulator through bipolar surface electrodes. The sensory nerve action potentials are recorded orthodromically with stimulating ring electrodes placed around the proximal and distal interphalangeal joints. Maximum sensory conduction velocity and maximum sensory action potential amplitude (SAPa) of the median (M3, middle finger-wrist; M4 ring fringer-wrist) and ulnar (U4 ring finger-wrist) nerves are determined. The difference between U4-M4 SCV is also calculated. Skin temperature was maintained > 32°C with an infrared lamp and recorded with a digital thermometer. Neurographic values at least 2 SD above or below the mean of the normative data of our laboratory (see below) are considered abnormal. Patients are eligible for the study if electrodiagnostic studies demonstrates any one of the following: I) abnormal comparative test i.e. a difference of >10 m/s between sensory conduction velocity of the median (M4) and ulnar (U4) nerves; II) abnormal digit/wrist sensory conduction velocity (M3 < 45 m/s and/or M4 < 43 m/s) and normal distal motor latency (DML <4.3 ms) of the median nerve. In other words, we will select only patients belonging to class I and II of the electrophysiological classification of CTS severity reported by Padua et al. [53], excluding subjects with absence of the sensory action potential or delayed DML or absence of the compound muscle action potential (CMAP) of the median nerve.

Finally, recruitment properties of the median nerve were studied by analyzing the relationship between the intensity of electrical stimulation and the size of motor and sensory responses, i.e. the input-output curve (I-O curve). This technique is capable to reveal focal conduction slowing in the median nerve, not detectable by conventional electrodiagnostic tests, in mild CTS patients [54,55]. In fact, the relationship between the stimulus intensity (input) and the size of the response (output) defines the characteristic of motor/sensory axon recruitment and, in addition to conventional parameters such as maximum amplitude, latency and maximum conduction velocity of a peripheral nerve, allows us to analyse the following variables: a) threshold value (the initial component), reflecting the most excitable axons; b) slope (the second component), indicating the recruitment efficiency (gain); c) plateau phase (the third component), reflecting the maximal size of CMAP, as well as the activation of axons that are ultimately recruited.

In order to determine the relationship between the intensity of electrical stimulation and the size of the median nerve motor response, the stimulating electrode is initially placed over the wrist, and its position adjusted until the site with the lowest threshold for eliciting a CMAP of 0.1 mV (baseline-negative peak) will be established. In order to determine median nerve (M3) SAPa we use the threshold that produced a SAPa of 1 uV; all sensory responses will be averaged. Stimulus intensities are increased in steps of 0.2 mA until the maximum (motor and sensory)-wave will be obtained. I-O relationship data will be fitted to a Boltzmann sigmoidal function by the Lavenberg-Marquard non linear least-mean-square algorithm [56]. Recruitment curves are constructed by normalizing stimulus currents and response amplitudes. This enabled comparison of individual I-O relationships. Parameters of the curves obtained before treatment will be compared with those obtained one, six and 1 year months later.

The same neurophysiologist will perform all electrodiagnostic tests at baseline and follow-ups.

### Ultrasonographic criteria

High-resolution ultrasonographic examination at wrist is a powerful tool in the diagnosis of compression mononeuropathies, particularly CTS [57,58] and allows to eliminate rheumatological pathology: arthritis, deforming arthrosis, flexor tenosynovitis, trigger finger.

In the uncertain situations standard rx is performed.

High-resolution ultrasonographic examination is performed by the same rheumatologist, experienced in musculoskeletal disorders, after electrodiagnostic test. A real-time scanner (Esaote Technos Mp) with a 5-10 MHz linear array transducer will used. Patients are seated in a chair with arms extended, hands resting in a horizontal supine position on the examination couch, and fingers semi-extended. It will perform longitudinal and transverse scans of the median nerve from the distal segment of the forearm to the tunnel outlet. The median nerve cross-section area (CSA) is measured at the tunnel inlet (just before the proximal margin of the flexor retinaculum) because the highest concordance with nerve conduction study is detected. CSA measurements are performed at the inner border of the thin surements hyperechoic rim of the nerve (perineurium) using the manual tracing technique. The weight of the probe is applied without additional pressure. The intra-observer reliability for nerve measurement has been tested previously and published elsewhere [59]. The same expert will perform all ultrasonographic examinations at baseline and during the follow-ups.

### Treatment

Patients are randomly allocated to one of two groups: (i) single cortisone (Triamcinolone acetonide 20 mg/1 ml, Triamvirgi, Fisiopharma), or (ii) single progesterone (Hydroxyprogesterone caproate 170 mg/1 ml, Lentogest, A.M.S.A.) echo-guided injection. If bilateral symptoms are present, only the hand the patient retains as having the most severe symptoms will be treated. The randomization is done with a dedicated script in MS Office Excel^®^. Since the injected substance can be easily identified by the treating rheumatologist, the only persons blinded to the treatment, beside the patient, will be the professionals performing the echography and the electrophysiology tests.

### Sample size

The main output parameter is the Symptom Severity Scale (SSS) score of the self-administered Boston Carpal Tunnel Questionnaire. The values reported in the literature assessing the cortisone treatment effect after 2 and 3 months are ranging between 1.37 - 2.3 (mean ± standard deviation: 1.6 ± 0.7). It has been estimated that a minimum number of n = 26.2 subjects for each group would be necessary to reveal a significant (α = 0.05) decrease in the SSS score from 1.6 to 2.0 with enough power (90%). If a drop-out of 4 subjects is considered, the minimum number of subjects required should be adjusted to no. = 30.

### Data analysis

The values for each recorded parameter (NCV, CSA, BQ, VAS) will be submitted to a two-way ANOVA, with the main factors TREATMENT (cortisol vs progesterone) and TIME (baseline, 1 month, 6 months, and 12 months after the injection). The significance level will be set at *p *< 0.05. Tukey's test post-hoc analysis will be performed when necessary, in order compensate for possible type I errors.

### Design of the trial

Sixty women with idiopathic mild CTS will be evaluated at before (baseline), 1, 6 and 12 months after injection.

The major outcome of this study is to determine that locally-injected progesterone may be more beneficial than cortisone in carpal tunnel syndrome at clinical level (SSS-BQ, VAS). Secondary outcome measures are:

- duration of experimental therapy, with a short (after 1 month) and long follow-up (after 6 to 12 months);

- improvement of electrodiagnostic and ultasonographic anomalies at various follow-up;

- correlation of the neurophysiologic and ultrasonographic data with clinical evaluation;

- comparison of the beneficial and harmful effects of the cortisone versus progesterone.

## The Limit

The hydroxyprogesterone caproate is a synthetic steroid hormone which possesses progestational activity in pregnancy to prevent preterm birth. It is a non selective agonist for the classical progesterone receptors because it is able to bind both to androgen and glucocorticoid receptors.

The absence of natural injectable progesterone commercially available could bias our results to some extent.

## Conclusion

Peripheral nerves are able to synthesize and metabolize neuroactive steroids, as progesterone, and are a target for these molecules, since they express classical and non-classical steroid receptors [46]. Progesterone modulate the expression of key transcription factors for Schwann cell function, regulate Schwann cell proliferation and promote the expression of myelin proteins involved in the maintenance of myelin multilamellar structure, such as myelin protein zero and peripheral myelin protein 22. These actions may result in the protection and regeneration of peripheral nerves affected by different form of pathological alterations. Indeed, progesterone is able to counteract biochemical, morphological and functional alterations of peripheral nerves in different experimental models of neuropathy, including the alterations caused by aging, diabetic neuropathy and physical injury.

In our case local corticosteroid injection for CTS is no effective treatment that can stop or reverse median nerve damage and progesterone could to represent really a new therapeutic approach. Therefore the main goal of our study is to show the neuroprotective effects of the progesterone at the level of the peripheral nervous system in humans and "mild" CTS represents a good model.

The results of this trial will be presented as soon as they are available.

## Competing interests

The authors declare that they have no competing interests.

## Authors' contributions

All co-authors participated in study design and read/approved the final manuscript.
